# Estimated Glomerular Filtration Rate, Proteinuria, and Risk of Cardiovascular Diseases and All-cause Mortality in Diabetic Population: a Community-based Cohort Study

**DOI:** 10.1038/s41598-017-17965-z

**Published:** 2017-12-20

**Authors:** Anxin Wang, Guojuan Chen, Yibin Cao, Xiaoxue Liu, Zhaoping Su, Yanxia Luo, Zhan Zhao, Xia Li, Shuohua Chen, Shouling Wu, Xiuhua Guo

**Affiliations:** 1 Department of Epidemiology and Health Statistics, School of Public Health, Capital Medical University, Beijing, China; 2 Beijing Municipal Key Laboratory of Clinical Epidemiology, Capital Medical University, Beijing, China; 3 Department of Neurology, Beijing Tiantan Hospital, Capital Medical University, Beijing, China; 4 Department of Neurology, Tangshan Gongren Hospital, North China University of Science and Technology, Tangshan, China; 5 Department of Cardiology, Tangshan People’s Hospital, North China University of Science and Technology, Tangshan, China; 6 State Key Lab. of Transducer Technology, Institute of Electronics, Chinese Academy of Sciences, Beijing, China; 7 University of Chinese Academy of Sciences, Beijing, China; 8 Department of Mathematics and Statistics, La Trobe University, Melbourne, Australia; 9 Department of Cardiology, Kailuan Hospital, North China University of Science and Technology, Tangshan, China

## Abstract

Data about associations between estimated glomerular filtration rate (eGFR) and proteinuria with cardiovascular diseases (CVDs) and all-cause mortality among diabetic population is less described. We aimed to describe these associations in Chinese diabetic population, and investigate the difference between sexes. The study was based on 8,301 diabetic participants in the Kailuan study, who was free of CVDs at baseline. We used Cox proportional hazard models to examine the associations of eGFR and proteinuria with CVDs and all-cause mortality. A stratified analysis by gender was performed. During a median follow-up of 8.05 years, 917 deaths and 813 incident CVDs occurred. Adjusted for all potential confounders, eGFR was associated with all-cause mortality, but not associated with incidence of CVDs. Compared to those with eGFR ≥ 90 ml/min/1.73 m^2^, Participants with eGFR <45 ml/min/1.73 m^2^ had 1.50 fold higher risk of all-cause mortality. Adjusted for all potential confounders, proteinuria was associated with risk of both CVDs and all-cause mortality. Additionally, the risk of all-cause mortality by proteinuria was greater in women than that in men. Both lower eGFR and proteinuria are independent risk factors for all-cause mortality in the Chinese diabetic population. Proteinuria conferred excessive risk for CVDs, and especially in women.

## Introduction

The prevalence of diabetes in China was 9.7%, accounting for 92.4 million adults^1^. Approximately 40% of them developed chronic kidney disease (CKD), commonly defined as glomerular filtration rate (GFR) of < 60 ml/min per 1.73 m^2^ and/or the presence of proteinuria^2^. Both diabetes and CKD can increase the risk of cardiovascular diseases (CVDs)^3^. Thus, diabetic individuals have greater risk for CVDs and death than no diabetic individuals due to the additional risk of CKD^4^. Most studies have demonstrated that decreased eGFR and proteinuria was risk factors for CVDs and mortality in general population and selected populations with heart failure, hypertension and existing CVDs^5–7^. But data for those associations is limited in the diabetic population. Additionally, few studies described the difference between men and women in CVDs and all-cause mortality by eGFR and proteinuria in diabetic population. Moreover, it was somewhat controversial that estimated GFR (eGFR) presented as an independent predictor for CVDs in the general population, especially in women^8,9^. Accordingly, we aimed to examine the relationships of eGFR and proteinuria with CVDs and all-cause mortality in a large prospective cohort of participants with diabetes, and investigate the difference between sexes.

## Results

### Baseline Characteristics

The baseline characteristics according to eGFR levels and the present of proteinuria are shown in Table 1. The mean age of the 8,301 diabetic participants at entry was 56.3 ± 10.5 years. Of them, 81.85% were men, 11.06% had proteinuria. The percentage of participants according to eGFR levels were as following: 28.45% for eGFR ≥ 90 ml/min/1.73 m^2^; 52.52% for eGFR of 60–89 ml/min/1.73 m^2^; 14.64% for eGFR of 45–60 ml/min/1.73 m^2^; 4.38% for eGFR <45 ml/min/1.73 m^2^.

**Table 1:** Participant characteristics at baseline, grouped by estimated glomerular filtration rate and proteinuria.

	eGFR(ml/min/1.73 m^2^)					Proteinuria		
	≥90 (n = 2362)	60–89 (n = 4360)	45–60 (n = 1215)	<45 (n = 364)	P for trend	No (n = 7383)	Yes (n = 918)	P
Age (years)	52.35(8.95)	56.85(10.17)	61.2(11.20)	59.77(12.61)	<0.01	56.16(10.47)	57.74(10.97)	<0.01
Gender (% Men)	2049(86.75)	3550(81.42)	918(75.56)	277(76.10)	<0.01	6031(81.69)	763(83.12)	0.29
Illiteracy/Primary education, n (%)	212(9.34)	534(12.77)	229(19.67)	67(19.09)	<0.01	905(12.76)	137(15.64)	0.03
Income level <800 (¥)	1972(86.87)	3555(85.03)	1009(86.61)	306(87.43)	0.91	6100(86.01)	742(84.90)	0.36
Current smoking, n (%)	772(33.76)	1334(31.49)	268(22.75)	88(24.79)	<0.01	2208(30.79)	254(28.67)	0.14
Current drinking, n (%)	838(36.64)	1383(32.63)	254(21.54)	77(21.69)	<0.01	2309(32.19)	243(27.43)	<0.01
Frequent physical activity, n (%)	291(12.81)	912(21.86)	267(23.00)	64(18.29)	<0.01	1360(19.20)	174(19.93)	0.25
High salt intake, n (%)	247(10.87)	461(11.04)	111(9.54)	40(11.43)	0.56	775(10.94)	84(9.61)	0.27
Family history of MI, n (%)	40(1.69)	92(2.11)	14(1.15)	3(0.82)	0.18	135(1.83)	14(1.53)	0.51
Family history of stroke, n (%)	92(3.90)	208(4.77)	51(4.20)	10(2.75)	0.85	325(4.40)	36(3.92)	0.50
Hypertension, n (%)	1304(55.21)	2684(61.56)	892(73.42)	269(73.90)	<0.01	4443(60.18)	706(76.91)	<0.01
Dyslipidemia, n (%)	1159(49.07)	2267(52.00)	668(54.98)	241(66.21)	<0.01	3774(51.12)	561(61.11)	<0.01
Atrial fibrillation, n (%)	6(0.25)	28(0.64)	15(1.23)	5(1.37)	<0.01	40(0.54)	14(1.53)	<0.01
Use of antihypertensive drugs, n (%)	316(13.38)	864(19.82)	325(26.75)	83(22.80)	<0.01	1366(18.5)	222(24.18)	<0.01
Use of lowering lipid drugs, n (%)	31(1.31)	94(2.16)	38(3.13)	12(3.30)	<0.01	150(2.03)	25(2.72)	0.17
Use of antidiabetes drugs, n (%)	408(17.27)	1125(25.80)	334(27.49)	90(24.73)	<0.01	1739(23.55)	218(23.75)	0.90
Resting heart rate (bpm)	78.51(11.73)	76.46(11.01)	76.80(11.72)	77.73(11.10)	<0.01	76.76(10.97)	80.33(13.72)	<0.01
FPG (mmol/L)	9.34(2.90)	9.34(3.06)	9.56(3.23)	9.76(3.29)	0.01	9.29(3.01)	10.22(3.28)	<0.01
BMI (kg/m^2^)	25.99(3.52)	26.20(3.46)	26.36(3.46)	26.21(3.52)	0.01	26.12(3.46)	26.52(3.66)	<0.01
SBP (mmHg)	135.51(20.22)	138.92(21.50)	145.75(23.37)	145.16(21.74)	<0.01	138.23(21.31)	147.12(23.28)	<0.01
DBP (mmHg)	85.75(11.56)	86.18(12.16)	88.24(12.78)	87.59(13.06)	<0.01	85.94(11.90)	90.29(13.43)	<0.01
TG (mmol/L)	2.20(1.88)	2.17(1.81)	2.27(1.71)	3.48(3.03)	<0.01	2.20(1.86)	2.68(2.18)	<0.01
TC (mmol/L)	5.16(1.30)	5.23(1.22)	5.16(1.41)	5.29(2.16)	<0.01	5.18(1.29)	5.37(1.59)	<0.01
LDL (mmol/L)	2.27(1.05)	2.46(0.98)	2.59(0.97)	2.58(0.87)	<0.01	2.43(0.99)	2.39(1.09)	0.34
HDL (mmol/L)	1.55(0.44)	1.55(0.43)	1.62(0.44)	1.62(0.46)	<0.01	1.56(0.43)	1.59(0.47)	0.05
UA (μmol/L)	261.99(81.79)	283.76(83.19)	292.88(85.49)	327.00(126.25)	<0.01	278.87(85.09)	296.22(97.67)	<0.01
CRP (mg/l)	3.39(8.20)	3.18(9.71)	3.09(6.96)	3.14(6.96)	0.33	3.04(8.42)	4.75(11.44)	<0.01
Scr (μmol/L)	68.88(13.85)	93.97(12.67)	117.62(14.77)	197.98(23.64)	<0.01	94.11(39.20)	100.78(42.86)	<0.01

Abbreviation: eGFR = estimated Glomerular Filtration Rate; MI = Myocardial Infarction; FPG = Fasting Plasma Glucose; BMI = Body Mass Index; SBP = Systolic Blood Pressure; DBP = Diastolic Blood Pressure; TG = Triglycerides; TC = Total Cholesterol; LDL = Low Density Lipoprotein; HDL = High Density Lipoprotein; UA = Uric Acid; CRP = C-Reactive Protein; Scr = Serum creatinine.

Compared with participants in the high eGFR categoty, participants with lower levels of eGFR were older, more likely to be women, had a higher education level; were less prevalent to smoke, drink and exercise; were more prevalent to have hypertension, dyslipidemia, atrial fibrillation, and use antihypertensive, antidiabetes, and lowering lipid drugs; had lower resting heart rate, body mass index (BMI) and low density lipoprotein (LDL); had higher fasting plasma glucose (FPG), systolic blood pressure (SBP), diastolic blood pressure (DBP), triglyceride (TG), total cholesterol (TC), high density lipoprotein (HDL), uric acid (UA) and creatinine (Cr) (all P for trend <0.05). Compared to participants without proteinuria, those with proteinuria were older, had a higher education level; were less prevalent to drink currently; were more prevalent to have hypertension, dyslipidemia, atrial fibrillation, and use antihypertensive drugs; had higher resting heart rate, FPG, BMI, SBP, DBP, TG, TC, UA, C-reactive protein (CRP) and Cr (all P < 0.05).

### Associations between eGRF and Proteinuria with CVDs and all-cause mortality

**Figure 1:**
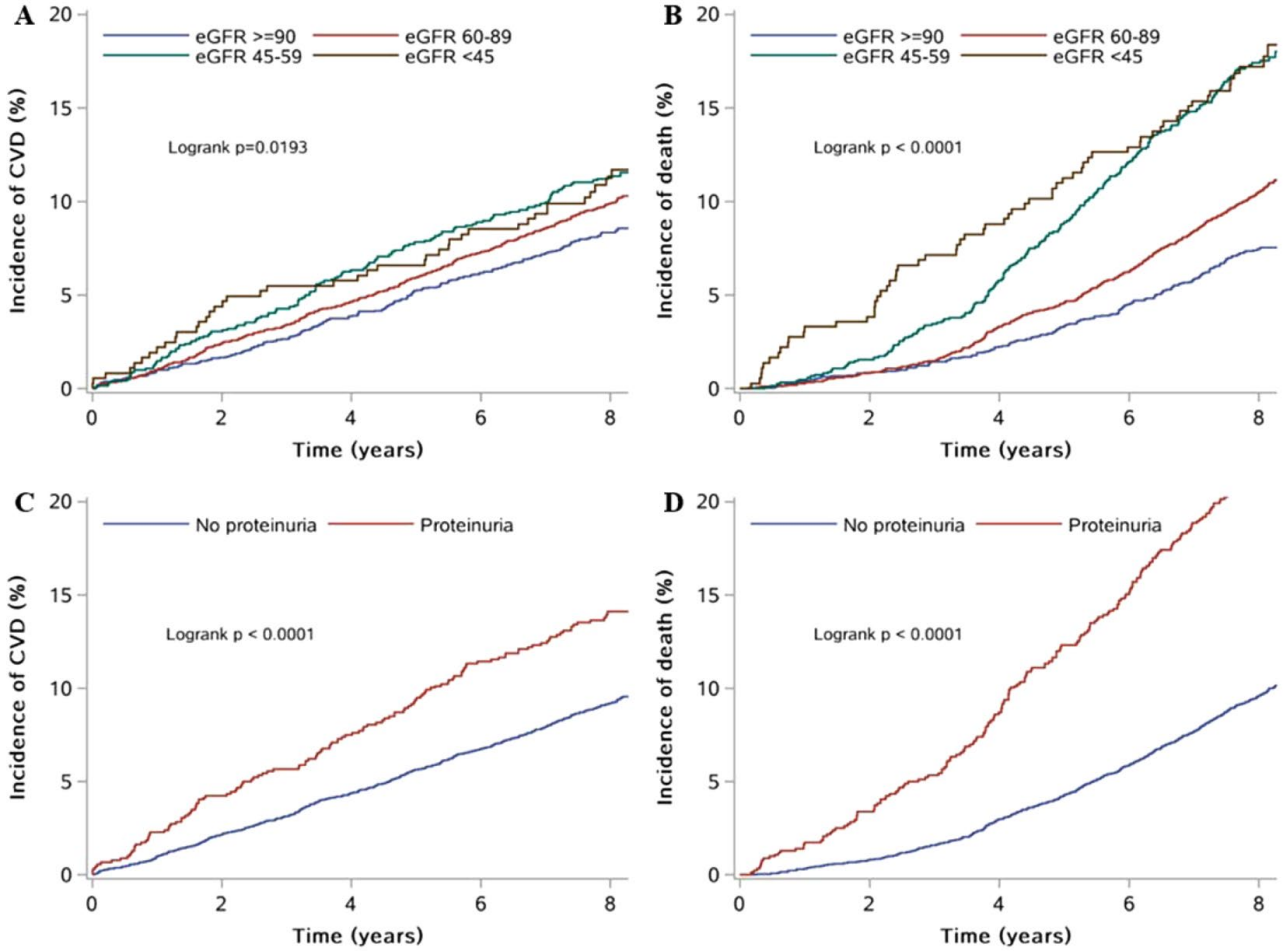
Kaplan-Meier curves of cumulative incidence of CVDs and all-cause mortality. (**A**) Is a Kaplan-Meier curve of incidence of CVDs according to categories of eGFR. (**B**) Is a Kaplan-Meier curve of incidence of all-cause mortality according to categories of eGFR. (**C**) is a Kaplan-Meier curve of incidence of CVDs according to the present of proteinuria. (**D**) Is a Kaplan-Meier curve of incidence of all-cause mortality according to categories of to the present of proteinuria. Abbreviation: eGFR = estimated Glomerular Filtration Rate; CVD = Cardiovascular Diseases.

The Kaplan-Meier curves are shown in Fig. 1. During a median follow-up of 8.05 years, 813 participants had CVDs, and 917 participants died. All the curves separate early and continue to diverge throughout the follow-up period. And participants with lower eGFR categories or proteinuria are more likely to have CVDs or die from all causes over time (log-rank test P < 0.01, for all).

Table 2 shows the association between eGFR and CVDs/all-cause mortality. The group of participants with eGFR of at least 90 ml/min/1.73 m^2^ was used as the reference group. In unadjusted regression analysis, the risk of CVDs increased sharply with eGFR declining (Crude Model). When adjusted, the relationship between eGFR and CVDs was no longer statistically significant (Model 1, Model 2 and Model 3). While, the risk of all-cause mortality increased with progressive decline of eGFR in both univariate and multivariate regression analyses (all P for trend < 0.05). In the full adjusted model (Model 3), participants with eGFR <45 ml/min/1.73 m^2^ had 1.5 fold higher risk of all-cause mortality (95%CI: 1.04–2.15), compared to participants with eGFR ≥ 90 ml/min/1.73 m^2^.

**Table 2:** Hazard ratios of cardiovascular diseases and all-cause mortality by estimated glomerular filtration rate and proteinuria.

	eGFR(ml/min/1.73 m^2^)					Proteinuria	
	≥90	60–89	45–60	<45	P for trend	No	Yes
**CVDs**							
No. of cases	197	437	138	41		685	128
Incidence rate/1000 person-year	10.8	13.1	15.0	14.9		12.1	18.8
Crude model	Ref.	1.37(1.14–1.64)	1.77(1.38–2.26)	1.86(1.30–2.67)	<0.01	Ref.	1.54(1.28–1.87)
Model 1	Ref.	1.11(0.92–1.34)	1.18(0.91–1.54)	1.24(0.85–1.81)	0.16	Ref.	1.44(1.19–1.74)
Model 2	Ref.	1.08(0.88–1.33)	1.04(0.78–1.39)	0.98(0.64–1.50)	0.97	Ref.	1.25(1.02–1.54)
Model 3	Ref.	1.08(0.88–1.32)	1.04(0.78–1.40)	1.00(0.63–1.50)	0.88	Ref.	1.27(1.03–1.56)
**All-cause mortality**							
No. of cases	175	465	213	64		716	201
Incidence rate/1000 person-year	9.5	13.8	23.4	24.1		12.5	30.2
Crude model	Ref.	1.86(1.54–2.24)	3.76(3.00–4.72)	4.41(3.23–6.02)	<0.01	Ref.	2.59(2.21–3.03)
Model 1	Ref.	1.10(0.90–1.33)	1.38(1.08–1.77)	1.52(1.10–2.11)	<0.01	Ref.	2.32(1.98–2.72)
Model 2	Ref.	1.04(0.84–1.27)	1.35(1.04–1.77)	1.66(1.17–2.37)	<0.01	Ref.	2.32(1.95–2.75)
Model 3	Ref.	1.03(0.84–1.27)	1.26(0.96–1.65)	1.50(1.04–2.15)	<0.01	Ref.	2.28(1.92–2.72)

Abbreviation: eGFR = estimated Glomerular Filtration Rate; CVD = Cardiovascular Diseases.

Model 1: adjusted for age, gender.

Model 2: adjusted for age, gender, education, income level, smoking status, drinking status, physical activity frequency, salt intake, family history of myocardial infarction, family history of stroke, hypertension, dyslipidemia, atrial fibrillation, use of antihypertensive drugs, use of lowering lipid drugs, use of antidiabetes drugs, resting heart rate, fasting plasma glucose, body mass index, systolic blood pressure, diastolic blood pressure, triglycerides, total cholesterol, low density lipoprotein cholesterol, and high density lipoprotein cholesterol.

Model 3: adjusted for the factors included in Model 2 plus uric acid, C-reactive protein, and serum creatinine.

Table 2 also shows the association between proteinuria and CVDs/all-cause mortality. Proteinuria significantly increased the risk of CVDs and all-cause mortality in both univariate and multivariate regression analyses. The fully adjusted hazard ratios (HRs) of CVDs and all-cause mortality were 1.27 (95% CI: 1.03–1.56, P < 0.01) and 2.28 (95% CI: 1.92–2.72, P < 0.01), respectively.

### Stratified Analysis by Gender

Table 3 shows the stratified analysis by gender for HRs of CVDs and all-cause mortality by eGFR and proteinuria. In the age-adjusted analysis, eGFR was not associated with risk of CVDs in both men and women. So as the multivariate analysis. While, the risk of all-cause mortality increased with eGFR declining in both men and women, whether in age-adjusted analysis or in multivariate analysis (all P for trend <0.05). Compared to participants with eGFR ≥ 90 ml/min/1.73 m^2^, participants with eGFR <45 ml/min/1.73 m^2^ had 1.47 fold higher full-adjusted risk of all-cause mortality in men (95%CI: 1.00–2.18), and 4.26 fold higher full-adjusted risk of all-cause mortality in women (95%CI: 1.38–13.19). However, gender showed no interaction terms with eGFR for CVDs or all-cause mortality (P interaction > 0.05, for both).

**Table 3:** Stratified analysis by gender for hazard ratios of cardiovascular diseases and all-cause mortality.

	eGFR (ml/min/1.73 m^2^)^a^					Proteinuria^b^	
	≥90	60–89	45–60	<45	P for trend	No	Yes
**Men**							
CVDs							
Age-adjusted	Ref.	1.16(0.95–1.42)	1.20(0.90–1.59)	1.18(0.78–1.79)	0.25	Ref.	1.36(1.11–1.68)
Multivariable	Ref.	1.12(0.90–1.39)	1.04(0.76–1.43)	1.03(0.64–1.65)	0.85	Ref.	1.18(0.94–1.48)
All-cause mortality							
Age-adjusted	Ref.	1.05(0.86–1.29)	1.31(1.00–1.70)	1.41(0.98–2.02)	0.01	Ref.	2.20(1.86–2.61)
Multivariable	Ref.	0.96(0.77–1.19)	1.21(0.91–1.61)	1.47(1.00–2.18)	0.03	Ref.	2.15(1.79–2.59)
**Women**							
CVDs							
Age-adjusted	Ref.	0.79(0.46–1.33)	1.13(0.57–2.25)	1.60(0.66–3.86)	0.29	Ref.	2.02(1.23–3.34)
Multivariable	Ref.	0.83(0.45–1.53)	1.41(0.61–3.25)	1.43(0.44–4.66)	0.36	Ref.	1.92(1.09–3.40)
All-cause mortality							
Age-adjusted	Ref.	1.72(0.85–3.47)	2.37(1.05–5.34)	3.04(1.17–7.91)	0.02	Ref.	3.48(2.23–5.45)
Multivariable	Ref.	2.20(0.99–4.88)	3.49(1.36–8.96)	4.26(1.38–13.19)_)	0.01	Ref.	3.96(2.39–6.57)

Abbreviation: eGFR = estimated Glomerular Filtration Rate; CVD = Cardiovascular Diseases.

^a^P interaction of gender and eGFR for CVDs is 0.26; P interaction of gender and eGFR for all-cause mortality is 0.64.

^b^P interaction of gender and proteinuria for CVDs is 0.08; P interaction of gender and proteinuria for all-cause mortality is 0.03.

^c^Adjusted for age, gender, education, income level, smoking status, drinking status, physical activity frequency, salt intake, family history of myocardial infarction, family history of stroke, hypertension, dyslipidemia, atrial fibrillation, use of antihypertensive drugs, use of lowering lipid drugs, use of antidiabetes drugs, resting heart rate, fasting plasma glucose, body mass index, systolic blood pressure, diastolic blood pressure, triglycerides, total cholesterol, low density lipoprotein cholesterol, high density lipoprotein cholesterol, uric acid, C-reactive protein, and serum creatinine.

In the age-adjusted analysis, proteinuria increased the risk of CVDs in both men and women. And in the multivariate analysis, the relationship remained statistically significant in women, but was no more significant in men. Compared to women without proteinuria, women with proteinuria had 1.92 fold full-adjusted risk of CVDs (95%CI: 1.09–3.40). While, whether in age-adjusted analysis or in multivariate analysis, proteinuria increased the risk of all-cause mortality in both men and women (all P < 0.05). Compared to participants without proteinuria, participants with proteinuria had 2.15 fold full-adjusted risk of all-cause mortality in men (95%CI: 1.79–2.59), and 3.96 fold full-adjusted risk of all-cause mortality in women (95%CI: 2.39–6.57). Gender showed no interaction terms with proteinuria for CVDs (P interaction = 0.08), but significant for all-cause mortality (P interaction = 0.03).

## Discussion

In this cohort of Chinese diabetic population, both lower eGFR and proteinuria were associated with all-cause mortality. There was no difference for all-cause mortality by eGFR between men and women. But women had greater risk for all-cause mortality by proteinuria than men. In this diabetic population, eGFR was not an independent risk factor for CVDs, but proteinuria was. There was no difference for this association by proteinuria between men and women.

Previous studies have established the prognostic role of eGFR changes for the cardiovascular mortality and all-cause mortality^10–17^. In our study, the result of the association between eGFR and all-cause mortality was consistent with previous ones. Most studies reported that eGFR was also associated with higher risk of stroke, myocardial infarction (MI), congestive heart failure, and composite CVDs. However, there was conflicting data as to the relationship of eGFR and CVDs in general population^7–9^. Some studies found that eGFR couldn’t predict the incidence of CVDs. In a large prospective cohort, Kurth T *et al*. observed no increase in risk of CVDs among women with less severe impairment of kidney function (defined by eGFR)^9,16^. In the current study, we demonstrated eGFR was not an independent predictor for CVDs in diabetic popualtion. Some researchers attribute the conflicting data in general population to positive studies including patients with existing CVDs or at high risk for CVDs^9^. But how to explain the different results in diabetic population? Also in the diabetic population, some studies found that reduced eGFR increased the risk of incident coronary heart disease and stroke^4,18^. Racial disparities were observed in the relationship between urinary albumin to creatinine ratio (ACR) and eGFR with incident stroke^7^. Higher ACR was independently associated with higher risk of stroke in black but not white participants. We hypothesized that racial disparities might account for the difference, because our study was conducted in Asians. Further studies are needed to confirm these findings and hypothesis.

In addition to the reduced eGFR, proteinuria is another important marker for kidney injury. It has been regarded as a strong predictor for death due to its significant relations with higher cerebrovascular risk and progressive renal damage. The associations between proteinuria with CVDs and death have been confirmed in general population^11,19,20^. In essential hypertensive patients, the associations tend to be more significant^21^. Proteinuria has been reported to increase the risk of CVDs and all-cause mortality in both non-diabetic and type 2 diabetic individuals^22,23^. But individuals with diabetes have a higher risk than those without^24^. However, the difference between men and women about this association was less explored before. In the present study, diabetic women with proteinuria had a higher risk of all-cause mortality than diabetic men (HR: 3.96 vs 2.15). The result was consistent with outcomes reported by Irie F *et al*. in Japanese general population: the multivariable relative risk of CVD death for positive vs negative proteinuria was 2.15 among women and 1.38 among men^25^. Similar associations were observed for all-cause mortality^25^. However, in the present study, only 1570 diabetic women were included and the number of incident CVDs and death was small. There may be some bias due to the low outcome incidence. Further studies are needed to confirm this finding.

Limitations of our study should be noted. First, the cohort participants were confined to coal workers in Tangshan city, thus our findings may not be generalized to other Chinese diabetic people. Second, using a single urine collection could be prone to measurement bias because proteinuria measured in a spot urine sample varies from day to day. Third, as mentioned before, there was a low outcome incidence among women participants, which might influence the result that women had a higher risk for all-cause mortality than men. Finally, despite adjusting over 20 confounders in the present study, there are still some unmeasured confounders that may influence the results but did not include. The strengths of this study include the large prospective cohort in diabetic population that allows precise estimations of the independent effect of reduced eGFR and proteinuria on the risk of CVDs and all-cause mortality among men and women.

## Conclusion

Our findings suggest that both low eGFR or proteinuria are predictors for all-cause mortality in the Chinese population. Additionally, diabetic patients with proteinuria are at high risk to develop CVDs. For persons with proteinuria, the mortality risk was higher in women than that in men. But this find needs further investigations.

## Methods

### Study Population

The study population came from the Kailuan study, which is a community-based, prospective cohort study designed to investigate the risk factors and prevention of CVDs in Chinese population. The study’s rationale, design and method have been described elsewhere^21,22^. Briefly, a total of 101,510 residents (aged ≥ 18 years; 81,110 men and 20,400 women) in the Kailuan community of Tangshan City were enrolled between June 2006 and October 2007. Information was collected by questionnaire assessment, clinical examination and laboratory assessment. 9,489 participants were diagnosed with diabetes: with FPG ≥ 7.0 mmol/L, or being diagnosed with type 2 diabetes before, or having used hypoglycemic drugs. Among them, the following participants were excluded: 32 participants who had a history of MI and/or stroke and 456 participants who lacked eGFR and urine dipstick protein assessments at baseline. Therefore, 8,301 participants including 6,794 men and 1,507 women were included in this study.

The study has been approved by the Ethics Committees of Kailuan General Hospital and Beijing Tiantan Hospital, following the guidelines outlined by the Helsinki Declaration. All participants provided written informed consent.

### Assessment of eGFR and Proteinuria

Blood and urine samples were both collected in the morning, following an overnight fast (>8 h). The eGFR was calculated from serum creatinine (Scr) using the Chinese coefficient-modified Chronic Kidney Disease Epidemiology Collaboration (CKD-EPI) formula^26^:

$${{\rm{eGFR}}}_{\mathrm{CKD}-\mathrm{EPI}}=141\times \,{\rm{\min }}\,{(\mathrm{SCr}/{\rm{\kappa }},1)}^{{\rm{\alpha }}}\times \,{\rm{\max }}\,{(\mathrm{SCr}/{\rm{\kappa }},1)}^{-1.209}\times {0.993}^{{\rm{Age}}}\times 1.018\,({\rm{if}}\,{\rm{female}})\times 1.1$$

where κ was 0.7 for females and 0.9 for males, α was −0.329 for females and −0.411 for males, min was the minimum of SCr/κ or 1, and max indicated the maximum of SCr/κ or 1. In this study, the eGFR values were divided into four levels: < 45, 45 to 59, 60 to 89, ≥90 mL/min/1.73 m^2^, based on National Kidney Foundation’s Kidney Disease Outcomes Quality Initiative^27^. Proteinuria was tested by urine dipstick. Test results were none, trace, 1+ , 2+ , and 3+ . We defined proteinuria as 1+ or greater protein in this study.

### Assessment of Potential Covariates

Demographic information was obtained through a questionnaire administrated by well-trained investigators.

Clinical examination and laboratory assessment were conducted at the 11 community hospitals, and analyzed at the central laboratory of the Kailuan General Hospital. Anthropomorphic parameters such as height, weight, waist, and blood pressure were measured with standard procedure, which have been described previously^28,29^. BMI was calculated as weight(kg)/height(m)^2^. Hypertension was defined as SBP ≥ 140 mmHg, or DBP ≥ 90 mmHg, or any use of antihypertensive drug, or self-reported history of hypertension. Dyslipidemia was defined as TC ≥ 150 mg/dl, or LDL ≥ 140 mg/dl, or HDL <40 mg/dl, or any use of lipid-lowering drugs, or self-reported history of dyslipidemia.

### Outcome Ascertainment

The outcome of interest was incidence of CVDs or death from all causes after baseline screening. CVDs here were defined as occurrence of stroke or MI. Specifically, stroke was diagnosed according to the World Health Organization criteria and confirmed by brain computed tomography or magnetic resonance. MI was diagnosed according to the World Health Organization criteria and confirmed by abnormal levels of cardiac enzymes or diagnostic electrocardiograms.

Participants were followed up by face-to-face interviews at the 11 community hospitals every 2 years. They self reported CVD events. Additional information of outcomes was confirmed by checking discharge summaries from the 11 hospitals and medical records from medical insurance. Subjects who didn’t participate the interview of the year would be checked for death from provincial vital statistics offices. Because all participants’ health insurance was covered by the Kailuan Medical Group, the impacts of loss to follow-up could be modest^28,29^.

### Statistical Analysis

Means, standard deviation, and percentages were used to describe baseline characteristics. The differences of baseline variables between proteinuria groups were tested by t test, or one-way ANOVA, or χ^2^ test, as appropriate. P for trend was calculated across eGFR categories. Kaplan-Meier method was used to calculate the cumulative incidence of CVDs and death over time. Multivariate Cox proportional hazard models were used to examine the associations of eGFR and proteinuria with CVDs and all-cause mortality. The full-model was adjusted for age, gender, education, income level, smoking status, drinking status, physical activity frequency, salt intake, family history of MI, family history of stroke, hypertension, dyslipidemia, atrial fibrillation, use of antihypertensive drugs, use of lowering lipid drugs, use of antidiabetic drugs, resting heart rate, FPG, BMI, SBP, DBP, TG, TC, LDL, HDL, UA, CRP, and Scr. Results were presented as HRs with 95% confidence interval (95% CI). Analyses were repeated after stratification by gender. Tests for trend were conducted in the eGFR categories. Values of P < 0.05 were accepted as indicative of statistical significance. All data was analyzed using the SAS version 9.3 (SAS Institute, Cary, North Carolina).

## References

[1] Yang W (2010). Prevalence of diabetes among men and women in China. N Engl J Med.

[2] K/DOQI clinical practice guidelines for chronic kidney disease: evaluation, classification, and stratification. *Am J Kidney Dis* **39**, S1–266 (2002). 11904577

[3] Rysz J (2007). Serum matrix metalloproteinases MMP-2 and MMP-9 and metalloproteinase tissue inhibitors TIMP-1 and TIMP-2 in diabetic nephropathy. J Nephrol.

[4] Wang Y (2014). Kidney function and the risk of cardiovascular disease in patients with type 2 diabetes. Kidney Int.

[5] Li Z (2015). Impact of proteinuria and glomerular filtration rate on risk of ischaemic and intracerebral hemorrhagic stroke: a result from the Kailuan study. Eur J Neurol.

[6] Mahmoodi BK (2014). Association of kidney disease measures with ischemic versus hemorrhagic strokes: pooled analyses of 4 prospective community-based cohorts. Stroke.

[7] Gutierrez OM (2012). Racial differences in albuminuria, kidney function, and risk of stroke. Neurology.

[8] Culleton BF (1999). Cardiovascular disease and mortality in a community-based cohort with mild renal insufficiency. Kidney Int.

[9] Kurth T, de Jong PE, Cook NR, Buring JE, Ridker PM (2009). Kidney function and risk of cardiovascular disease and mortality in women: a prospective cohort study. BMJ.

[10] Arce CM (2016). Kidney Function and Cardiovascular Events in Postmenopausal Women: The Impact of Race and Ethnicity in the Women’s Health Initiative. Am J Kidney Dis.

[11] Astor BC, Hallan SI, Miller ER, Yeung E, Coresh J (2008). Glomerular filtration rate, albuminuria, and risk of cardiovascular and all-cause mortality in the US population. Am J Epidemiol.

[12] Go AS, Chertow GM, Fan D, McCulloch CE, Hsu CY (2004). Chronic kidney disease and the risks of death, cardiovascular events, and hospitalization. N Engl J Med.

[13] Muntner P, He J, Hamm L, Loria C, Whelton PK (2002). Renal insufficiency and subsequent death resulting from cardiovascular disease in the United States. J Am Soc Nephrol.

[14] Franczyk-Skora B (2015). Sudden cardiac death in CKD patients. Int Urol Nephrol.

[15] Malyszko J, Banach M (2014). Pre-CKD- do we need another hero?. Curr Vasc Pharmacol.

[16] Rysz J (2009). Nephroprotective and clinical potential of statins in dialyzed patients. Expert Opin Ther Targets.

[17] Colantonio LD (2015). Contrasting Cholesterol Management Guidelines for Adults with CKD. J Am Soc Nephrol.

[18] Ninomiya T (2009). Albuminuria and kidney function independently predict cardiovascular and renal outcomes in diabetes. J Am Soc Nephrol.

[19] Madison JR (2006). Proteinuria and risk for stroke and coronary heart disease during 27 years of follow-up: the Honolulu Heart Program. Arch Intern Med.

[20] Arnlov J (2005). Low-grade albuminuria and incidence of cardiovascular disease events in nonhypertensive and nondiabetic individuals: the Framingham Heart Study. Circulation.

[21] Schrader J (2006). Microalbuminuria and tubular proteinuria as risk predictors of cardiovascular morbidity and mortality in essential hypertension: final results of a prospective long-term study (MARPLE Study)*. J Hypertens.

[22] Berhane AM, Weil EJ, Knowler WC, Nelson RG, Hanson RL (2011). Albuminuria and estimated glomerular filtration rate as predictors of diabetic end-stage renal disease and death. Clin J Am Soc Nephrol.

[23] Sukhija R (2006). Relation of microalbuminuria and coronary artery disease in patients with and without diabetes mellitus. Am J Cardiol.

[24] Fox CS (2012). Associations of kidney disease measures with mortality and end-stage renal disease in individuals with and without diabetes: a meta-analysis. Lancet.

[25] Irie F (2006). The relationships of proteinuria, serum creatinine, glomerular filtration rate with cardiovascular disease mortality in Japanese general population. Kidney Int.

[26] Teo BW (2011). GFR estimating equations in a multiethnic Asian population. Am J Kidney Dis.

[27] Levey AS (2011). The definition, classification, and prognosis of chronic kidney disease: a KDIGO Controversies Conference report. Kidney Int.

[28] Wu S (2012). Intra-individual variability of high-sensitivity C-reactive protein in Chinese general population. Int J Cardiol.

[29] Wang A (2013). Measures of adiposity and risk of stroke in China: a result from the Kailuan study. PLoS One.

